# Biochar and Trehalose Co-Application: A Sustainable Strategy for Alleviating Lead Toxicity in Rice

**DOI:** 10.3390/plants14060878

**Published:** 2025-03-11

**Authors:** Yingfen Yang, Li Liu, Haibo Xiong, Tianju Wang, Jun Yang, Wenpeng Wang, Areej A. Al-Khalaf, Zhuhua Wang, Waqar Ahmed

**Affiliations:** 1 Academy of Science and Technology, Chuxiong Normal University, Chuxiong 675000, China; yangyf871@163.com (Y.Y.);; 2 College of Big Data, Yunnan Agricultural University, Kunming 650201, China; 3 College of Resources, Environment, and Chemistry, Chuxiong Normal University, Chuxiong 675000, China; 4 Biology Department, College of Science, Princess Nourah bint Abdulrahman University, Riyadh 11671, Saudi Arabia; 5 Guangdong Province Key Laboratory of Microbial Signals and Disease Control, College of Plant Protection, South China Agricultural University, Guangzhou 510642, China

**Keywords:** antioxidants, biochar, bioavailability, soil remediation, translocation factor

## Abstract

Lead (Pb) is a common contaminant that causes serious health and environmental problems. Thus, appropriate environmentally friendly and efficient techniques must be developed to remediate Pb in soils. Biochar (BC) has shown promise as an effective strategy to mitigate Pb toxicity. Trehalose (Tre) is a promising sugar that has been shown to effectively improve plant tolerance to abiotic stresses. Nonetheless, its role in alleviating Pb toxicity is unknown. The study investigated the impacts of BC and Tre co-application in alleviating Pb toxicity in rice crops. The study included the following treatments: control, Pb stress (250 mg kg^−1^), Pb stress (250 mg kg^−1^) + BC (2.5%), Pb stress (250 mg kg^−1^) + Tre (30 mM), and Pb stress (250 mg kg^−1^) + BC (2.5%) + Tre (30 mM). Results showed that Pb toxicity reduced rice yield by decreasing chlorophyll synthesis and relative water content (RWC), by increasing malondialdehyde (MDA) and hydrogen peroxide (H_2_O_2_) contents, Pb accumulation in roots and shoots, soil available Pb concentration, and by decreasing the availability of soil nutrients. BC and Tre application mitigated the adverse impacts of Pb; however, more promising results were obtained with the co-application of BC and Tre. The results indicated that co-application of BC and Tre increased the rice yield by increasing photosynthetic pigments (46–96.42%), leaf water contents (16.67%), proline and soluble protein synthesis (35.13% and 24.96%), and antioxidant activities (12.07–31.67%), by decreasing root (59.72%), shoot (76.47%), and soil (57.14%) Pb concentrations, and the Pb translocation factor (15.08%). These findings suggested that co-application of BC and Tre can be a practical approach for reducing Pb toxicity, availability, and uptake, which improves rice productivity in Pb-polluted soil.

## 1. Introduction

Heavy metal pollution is a serious threat to humans, plants, and the environment [1]. Heavy metals (HMs) are rapidly increasing in soils because of industrial effluents, mining, coal burning, fertilizers, pesticides, and the use of wastewater [1,2]. Industrial development has increased the concentration of HMs, including lead (Pb), causing ecosystem disturbance and groundwater pollution [3,4]. Lead is the second most dangerous metal [5], and it is considered a strong environmental pollutant by the Environment Protection Agency [5]. It exists in the environment due to human activities and poses a serious threat to all organisms [6]. The use of Pb-contaminated food is a way for Pb to enter into humans, where it causes damage to the liver, lungs, kidneys, reproductive system, bones, and brain [7].

Lead is a non-essential metal for plants; however, when plants absorb it, it reduces growth, biomass production, and nutrient uptake and causes the oxidation of lipids, proteins, and DNA by increasing the production of reactive oxygen species [7,8,9,10]. It also decreases transpiration and photosynthetic rates, chlorophyll synthesis, and stomatal conductance, and damages the photosynthetic apparatus, resulting in significant growth losses [11]. Pb also binds to the mitochondrial membrane and decouples the phosphorylation process, and disturbs the electron transport chain [12]. Moreover, it also induces the accumulation of NAD^+^-malate, increases dehydrogenase activity, and interrupts the Hill reaction, leading to a reduction in photosynthetic efficiency and subsequent plant growth [13,14]. Therefore, it is necessary to find ways to reduce Pb uptake by plants and its subsequent entry into humans.

Globally, diverse strategies are employed for the remediation of polluted soils. Biochar (BC) effectively reduces the bioavailability of HMs, including Pb [15,16]. BC immobilizes Pb in water and soil because of its large surface area and aromatic and heterocyclic carbon functional groups [16]. BC application improves soil organic matter, nutrient availability, and microbial activities [17,18], and it has excellent adsorption capacity, which helps to mitigate the toxicity of HMs [19]. Biochar increases plant growth by mitigating ROS and MDA production and increasing antioxidant activity and Pb immobilization [20]. Recent findings have shown that BC application enhances the growth and biomass production of rice by improving soil properties, antioxidant activities, and nutrient uptake, as well as reducing Pb availability and accumulation [21]. Trehalose (Tre) is a non-reducing sugar with appreciable potential for mitigating the toxic impacts of stress conditions [21]. It improves plant development and response to stress conditions [22,23]. Exogenous Tre applied to plants reduces sodium accumulation and prevents chlorophyll loss in plants facing saline conditions [24]. It also increases antioxidant activities, which ensures better plant growth [25]. Recent findings have shown that exogenous Tre increases antioxidant activities and reduces the mobility and toxicity of cadmium in rice plants, thereby improving rice growth [26].

No information is available in the literature about the co-application of BC and Tre to mitigate Pb toxicity and increase Pb immobilization. Thus, considering the above fact, we hypothesized that the co-application of BC and Tre could give better results in addressing Pb toxicity in rice than their individual application. The objectives of this study were to (i) explore the impacts of BC and Tre on the growth and physiology of rice and (ii) to determine the efficiency of BC and Tre in mitigating Pb bioavailability and improving soil properties.

## 2. Results

### 2.1. Growth and Yield Traits

**Figure 1:**
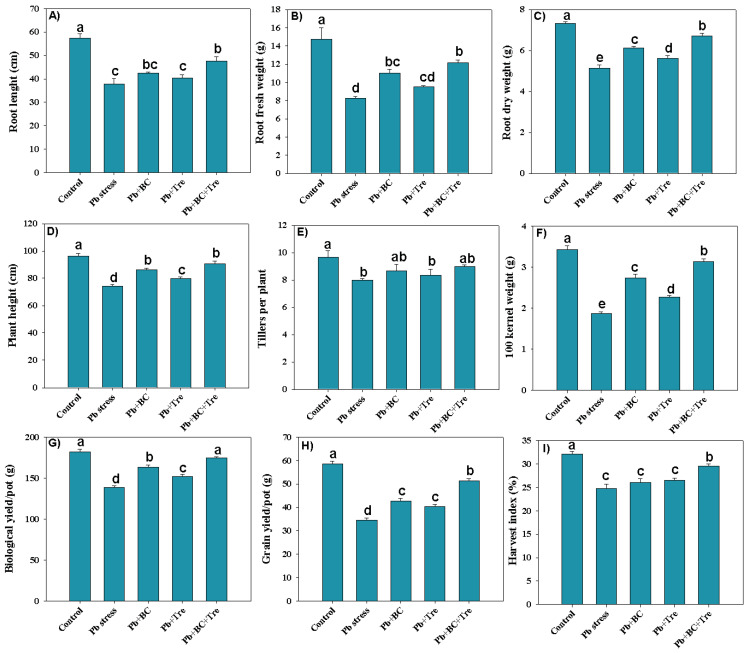
Effects of biochar and trehalose on growth and yield traits of rice plants under lead stress. (**A**) root length, (**B**) root fresh weight, (**C**) root dry weight, (**D**) plant height, (**E**) tillers per plant, (**F**) 100 kernel weight, (**G**) biological yield/pot, (**H**) grain yield/pot, and (**I**) harvest index. The data are presented as means ± SD (*n* = 3), and different letters indicate significance at *p* < 0.05 level according to the Tukey HSD test. Here, control (without Pb stress and BC), Pb stress (250 mg kg^−1^), Pb stress (250 mg kg^−1^) + BC (2.5%), Pb stress (250 mg kg^−1^) + Tre (30 mM), and Pb stress (250 mg kg^−1^) + BC (2.5%) + Tre (30 mM).

The data revealed that root length (RL), root fresh weight (RFW), and root dry weight (RDW) decreased by 52.64%, 78.20%, and 42.41%, respectively, under Pb stress (Figure 1A–C). The applied amendments markedly increased RL, RFW, and RDW, and co-application of BC and Tre increased RL, RFW, and RDW by 26.54%, 47.45%, and 30.54%, respectively, under Pb stress conditions (Figure 1A–C). The plant height (PH), number of tillers (TPP), and 100-kernel weight (100-KW) significantly decreased under Pb stress. However, BC and Tre reversed the toxic impacts of Pb and increased PH, TPP, and 100-KW. The combined addition of BC and Tre increased the pH, TPP, and 100-KW by 21.92%, 12.5%, and 67.38%, respectively, under Pb stress conditions (Figure 1D–F). Rice, biological yield (BY), grain yield (GY), and harvest index (HI) significantly decreased by 30.97%, 69.71%, and 29.58% under Pb stress, respectively. The applied amendments enhanced the BY, GY, and HI under Pb stress conditions. The co-application of BC + Tre increased BY, GY, and HI by 25.49%, 49.20%, and 18.86%, respectively, while BC increased BY, GY, and HI by 17.77%, 23.64%, and 4.95% respectively, and Tre increased BY, GY, and HI by 9.39%, 16.82%, and 6.77% respectively, under Pb stress conditions (Figure 1G–I).

### 2.2. Chlorophyll and Relative Water Contents

**Figure 2:**
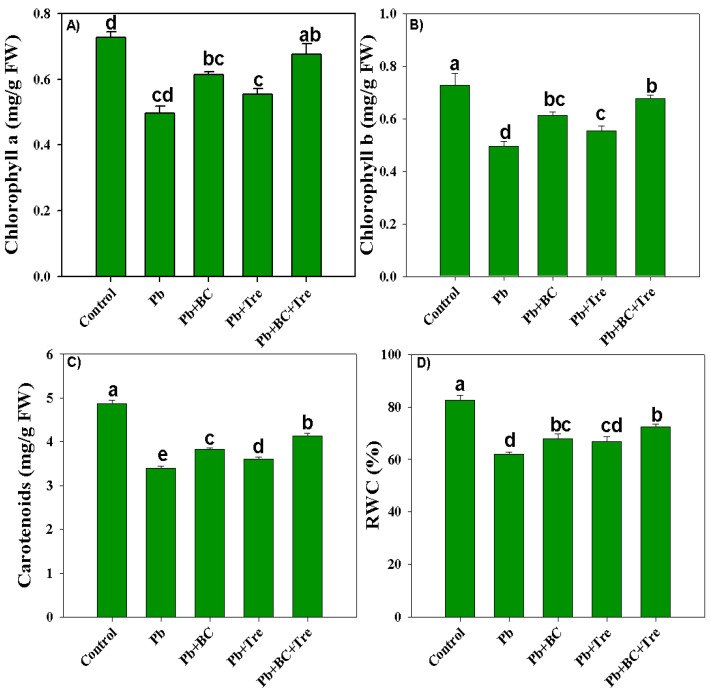
Effects of biochar and trehalose on photosynthetic pigments and RWC of rice under lead stress. (**A**) chlorophyll a, (**B**) chlorophyll b, (**C**) carotenoids, and (**D**) RWC. The data are presented as means ± SD (*n* = 3), and different letters indicate significance at *p* < 0.05 level according to the Tukey HSD test. RWC: relative water content. Here, control (without Pb stress and BC), Pb stress (250 mg kg^−1^), Pb stress (250 mg kg^−1^) + BC (2.5%), Pb stress (250 mg kg^−1^) + Tre (30 mM), and Pb stress (250 mg kg^−1^) + BC (2.5%) + Tre (30 mM).

Lead toxicity reduced the chlorophyll-a and chlorophyll-b contents by 46% and 96.42%, respectively. BC and Tre significantly increased the synthesis of chlorophyll-a and chlorophyll-b. Maximum increases of 41.67% and 57.14% in chlorophyll-a and chlorophyll-b, respectively, were noted with BC and Tre under Pb stress conditions (Figure 2A,B). Pb stress decreased the carotenoid concentration in the rice plants. A substantial decrease of 43.65% in carotenoid concentration was noted under Pb stress compared to control (Figure 2C). BC and Tre reversed Pb toxicity and improved carotenoid synthesis in the rice plants. The combined application of BC and Tre increased carotenoid contents by 21.82%, whereas BC and Tre addition increased carotenoid contents by 12.68% and 6.48%, respectively, under Pb stress conditions (Figure 2C). The leaf’s relative water content (RWC) significantly decreased by 33.33% under Pb stress. However, the co-application of BC and Tre significantly increased the leaf RWC compared with BC or Tre application alone (Figure 2D).

### 2.3. Leaf Oxidative Markers, Osmolytes, and Antioxidant Activities

Leaf oxidative stress markers were significantly increased under Pb stress. The results presented an increase of 148.26%, 85.46%, and 162% in electrolyte leakage (EL), malondialdehyde (MDA), and hydrogen peroxide (H_2_O_2_) under Pb stress, respectively, compared to control (Table 1). Briefly, co-application of BC and Tre significantly decreased EL, MDA, and H_2_O_2_ by 122.20%, 75.27%, and 70.54%, respectively under Pb stress. The amendments, PB and Tre, also decreased the EL, MDA, and H_2_O_2_; however, both had comparable effects in reducing the production of these oxidative markers (Table 1). Osmolyte synthesis showed a divergent response under stress conditions. The synthesis of proline was increased under Pb toxicity, whereas the total soluble protein (TSP) content decreased due to Pb toxicity (Table 1). Co-application of BC and Tre, BC, and Tre increased the proline concentration by 35.13%, 24.45%, and 8.34%, respectively, whereas co-application of BC and Tre, BC, and Tre increased the TSP by 24.96%, 17.96%, and 7.15%, respectively, under Pb stress conditions (Table 1). The results revealed that ascorbate peroxidase (APX), catalase (CAT), peroxidase (POD), and superoxide dismutase (SOD) activities increased under Pb stress indicating that plants activated their defense system to mitigate Pb toxicity. The results revealed that APX, CAT, POD, and SOD activities increased by 12.07%, 17.71%, 31.67%, and 21.09%, respectively, in rice leaves under Pb stress compared to control (Table 1). BC and Tre also increased antioxidant activities. However, their combined application showed better results than their individual applications did.

### 2.4. Tissue Pb Concentration, Pb Translocation, and Biological Accumulation Coefficient Factors

**Figure 3:**
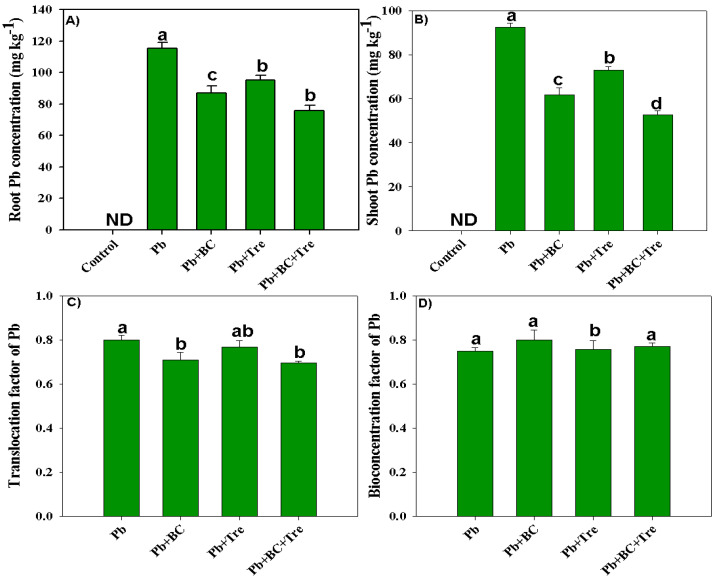
Effects of biochar and trehalose on root and shoot Pb concentration, translocation, and bio-concentration factor of Pb. (**A**) root Pb concentration, (**B**) shoot Pb concentration, (**C**) translocation factor of Pb, and (**D**) bioconcentration factor of Pb. The data are presented as means ± SD (*n* = 3), and different letters indicate significance at *p* < 0.05 level according to the Tukey HSD test. Here, control (without Pb stress and BC), Pb stress (250 mg kg^−1^), Pb stress (250 mg kg^−1^) + BC (2.5%), Pb stress (250 mg kg^−1^) + Tre (30 mM), and Pb stress (250 mg kg^−1^) + BC (2.5%) + Tre (30 mM).

The results revealed significantly higher Pb concentrations in the plant parts of the Pb-polluted soil (Figure 3). No Pb concentration was detected in control plants because Pb-free soil was used in the control (Figure 3). Concentrations of 115 and 92 mg kg^−1^ Pb were noted in roots and shoots, respectively (Figure 3A,B). All amendments decreased the Pb concentration, and the lowest concentrations in the roots (75.67 mg kg^−1^) and shoots (52.68 mg kg^−1^) were associated with the co-application of BC and Tre (Figure 3A,B). BC and Tre application significantly decreased the translocation factor (TF) and bio-concentration factor (BCF: Figure 3C,D), and the highest reductions in TF and BCF were obtained with the co-application of BC + Tre, followed by BC and Tre alone (Figure 3C,D).

### 2.5. Tissue Nutrient Concentration

The results indicated that Pb stress reduced the concentrations of all the studied nutrients in the rice roots and shoots. However, BC and Tre application mitigated the adverse impacts of Pb and increased the accumulation of all the nutrients in roots and shoots (Table 2). The results indicated that the maximum root (15.17 mg kg^−1^) and shoot nitrogen (N; 19.22 mg kg^−1^) contents were observed with combined BC and Tre application in Pb-polluted soil, and the lowest root and shoot N concentrations were observed in Pb-contaminated soil without amendment (Table 2). The co-application of BC and Tre increased the root phosphorus (*p*) and potassium (K) contents by 85.17% and 88.33%, respectively. In contrast, the same treatment increased the shoot P and K concentrations by 72.31% and 98%, respectively (Table 2). The levels of calcium (Ca) and magnesium (Mg) in both roots and shoots also decreased under Pb-polluted soil (Table 2). Nevertheless, BC and Tre alone and combined significantly enhanced the Ca and Mg concentrations. Co-application of BC and Tre increased the root Ca and Mg concentrations by 50.74% and 82.30%, respectively, and the same treatment combination increased the shoot Ca and Mg concentrations by 41.77% and 66%, respectively (Table 2).

### 2.6. Soil Properties After Harvesting

The results indicated a significant impact of Pb stress and applied treatments on soil properties after harvesting the rice crop (Table 3). The maximum soil Pb (154 mg kg^−1^) concentration was observed in Pb-polluted soil, while the combined application of BC and Tre reduced the soil Pb availability by 57.14% (Table 3). BC caused a slight increase in soil pH; nonetheless, Tre had a non-significant effect on soil pH (Table 3). The concentrations of available phosphorus (AP), available potassium (AK), and available nitrogen (TN) also markedly decreased under Pb stress conditions; however, all the treatments increased soil AP, AK, and AN. Maximum increases of 54.85%, 32.82%, and 47.56% in soil AP, AK, and TN, respectively, were observed with the co-application of BC and Tre under Pb stress conditions (Table 3). The results showed that only BC had a significant effect on SOC and a maximum increase of 35.04% in SOC was observed with BC + Tre combined application under Pb stress conditions (Table 3).

### 2.7. Principal Component Analysis and Correlation Matrix

**Figure 4:**
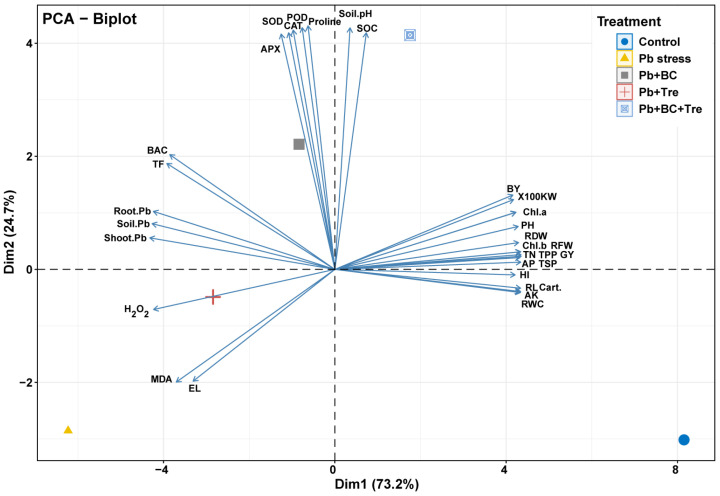
Principal component analysis of the impact of BC and Tre on rice under Pb stress conditions. The biplot of PCA indicates the interactions among different traits with diverse treatments RL: root length, RFW: root fresh weight, RDW: root dry weight, PH: plant height, TPP: tillers/plant, GW: grain weight, BY: biological yield, GY: grain yield, HI: harvest index, Chl: chlorophyll, Cart. carotenoids, RWC: relative water contents, EL: electrolyte leakage, MDA: malondialdehyde, H_2_O_2_: hydrogen peroxide, TSP: total soluble protein, APX: ascorbate peroxidase, CAT: catalase, POD: peroxidase, SOD: superoxide dismutase, AP: available phosphorus, AK: available potassium, TN: total nitrogen, SOC: soil organic carbon, TF: translocation factor, BAC: biological accumulation coefficient. Here, control (without Pb stress and BC), Pb stress (250 mg kg^−1^), Pb stress (250 mg kg^−1^) + BC (2.5%), Pb stress (250 mg kg^−1^) + Tre (30 mM), and Pb stress (250 mg kg^−1^) + BC (2.5%) + Tre (30 mM).

**Figure 5:**
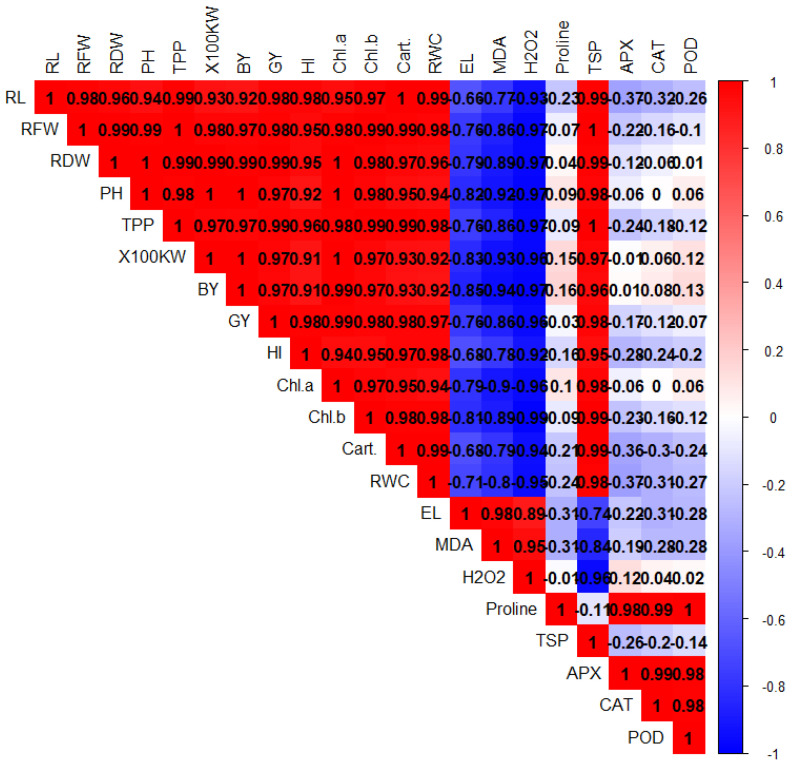
The correlation matrix for the effects of BC and Tre application on the growth, yield, and physiological traits of rice under Pb stress. The dark blue, light blue, and light red colors indicate positive relationships, whereas the dark red color indicates negative relationships. Here, RL: root length, RFW: root fresh weight, RDW: root dry weight, PH: plant height, TPP: tillers/plant, GW: grain weight, BY: biological yield, GY: grain yield, HI: harvest index, Chl: chlorophyll, Cart. carotenoids, RWC: relative water content, EL: electrolyte leakage, MDA: malondialdehyde, H_2_O_2_: hydrogen peroxide, TSP: total soluble protein, APX: ascorbate peroxidase, CAT: catalase, POD: peroxidase, SOD: superoxide dismutase.

**Figure 6:**
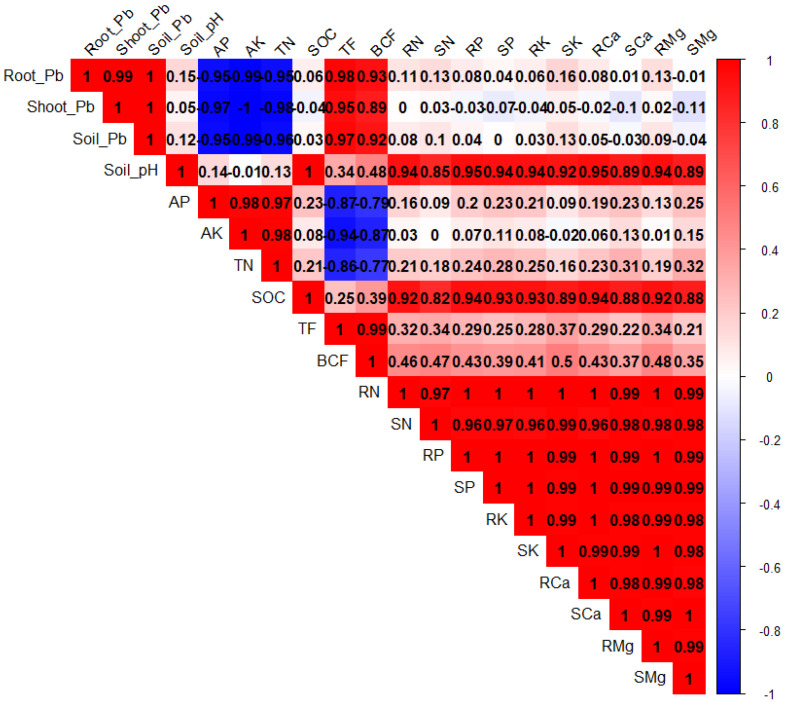
The correlation matrix for the effects of BC and Tre application on soil properties and tissue nutrient concentrations. The dark blue, light blue, and light red colors indicate positive relationships, whereas the dark red color indicates negative relationships. Here, AP: available phosphorus, AK: available potassium, TN: total nitrogen, SOC: soil organic carbon, TF: translocation factor, BCF: bio-concentration factor, RN: root nitrogen, SN: shoot nitrogen, RP: root phosphorus, SP: shoot phosphorous, RK: root potassium, SK: shoot potassium, RCa: root calcium, SCa: shoot calcium, RMg: root magnesium, SMg: shoot magnesium.

PCA was performed to evaluate the associations among different plant treatments after BC and Tre application under Pb stress (Figure 4). Both dimensions of PCA (Dim-1 and Dim-2) showed a variability of 97.9% in the dataset. The results also showed that Dim-1 has a share of 73.2%, and Dim-2 contributes 24.7%. BC and Tre significantly affected the studied traits. MDA, H_2_O_2_, and RWC contributed to Dim-1, and all the other studied traits were associated with Dim-2. These results were further determined via correlation matrix analysis (Figure 5 and Figure 6). The results revealed that MDA, EL, H_2_O_2_, soil-Pb, root and shoot Pb, BAC, and TF were positively associated with soil pH, SOC, proline, APX, CAT, POD, and SOD (Figure 5 and Figure 6). Moreover, MDA, EL, H_2_O_2_, soil-Pb, root and shoot Pb, BAC, and TF were negatively linked with RDW, chl-a, pH, grain weight, BY, GY, AP, RWC, RL, carotenoid, TN, TSP, TPP, and RFW (Figure 5 and Figure 6).

## 3. Discussion

Lead toxicity reduces rice growth and yield by decreasing photosynthetic pigments, leaf water status, soil nutrient availability, increasing oxidative damage, and Pb accumulation in plant tissues [9]. These findings align with previous studies that Pb toxicity decreased rice growth by causing oxidative damage, decreasing photosynthetic pigments, and disturbing antioxidant activities [27]. Leaf photosynthetic pigments are important indicators for detecting the impacts of toxic metals on plants [28,29]. Lead toxicity decreases the photosynthetic pigments, which is linked to the increased activity of chlorophyll-degrading enzymes [30]. Pb stress increases oxidative damage and decreases nutrient uptake (iron and magnesium), reducing chlorophyll synthesis and subsequent plant growth [12,31]. These findings are consistent with previous studies indicating that Pb toxicity decreases chlorophyll synthesis and subsequent plant growth by impairing chlorophyll synthesis [32]. Pb toxicity also inhibits cell division, damages chloroplasts, and restricts electron transport in the Calvin cycle, leading to poor plant growth [33,34].

Abiotic stresses increase oxidative damage by increasing MDA and H_2_O_2_ production, which reduces photosynthetic efficiency and leads to poor and stunted growth [9]. The Pb-induced increase in MDA and H_2_O_2_ production caused membrane damage, which was characterized by a substantial increase in EL from the leaves. Previously, different authors reported that Pb toxicity in rice and *Conocarpus erectus* significantly increased MDA, H_2_O_2_, and EL contents [35,36]. We also noted that Pb stress significantly affected leaf water status, which could be attributed to Pb-induced water stress, which caused a reduction in water availability and, subsequently, leaf water content [9].

Biochar and Tre enhanced rice growth and yield traits associated with increased chlorophyll synthesis, antioxidant activity, nutrient uptake, and SOC and decreased Pb availability. The application of both BC and Tre increased the photosynthetic pigments and RWC, and reduced the MDA and H_2_O_2_ contents by increasing the antioxidant activity and synthesis of potential osmolytes, which increased rice growth and production [27,28,37]. These findings corroborate earlier studies indicating that BC and Tre application improves plant growth under stress conditions by decreasing damage and increasing antioxidant activity [27,28,38]. We also hypothesized that Tre might form Tre–Pb complexes, reducing Pb uptake and improving plant growth. Trehalose application also enhanced the photosynthetic pigments, indicating that Tre protected the apparatus, thereby increasing plant growth [39]. The foliar spray of Tre also reduced oxidative stress by decreasing MDA and H_2_O_2_ production, which was linked to improved antioxidant activities and increased synthesis of proline and TSP. Trehalose also participates in stress signaling and triggers antioxidant activity and osmolyte production, which help counteract the toxic impacts of metals [40]. The increase in TSP synthesis can be attributed to Tre’s ability to stabilize proteins and enzymes involved in the synthesis of proteins [41].

Lead toxicity strongly reduced the nutrient concentration in the tissue, whereas the different treatments significantly increased the nutrient concentration in the tissue. Pb interferes with the transport of essential nutrients and disrupts the metabolic pathways and soil microbial activity, which are crucial for nutrient availability, thereby leading to lower nutrient concentration in plant parts [29]. These findings align with the study of Jasmin et al. [42], who reported that Pb toxicity severely reduced nutrient uptake by rice plants grown in Pb-contaminated soil. BC and Tre increased the tissue nutrient concentration. BC application enhances SOC, which fixes Pb in soil and decreases its availability, reducing the competition of Pb with nutrient uptake, thereby increasing nutrient uptake [31]. Further, BC and Tre also increased the root growth and surface area, and both BC and Tre might also maintain better osmotic regulation, which maintained turgor pressure, thereby increasing the ability of rice plants to take up more water and nutrients. Previously, different authors reported that BC application improved nutrient uptake, favoring plant growth under heavy metal stress [20,43]. Likewise, Kosar et al. [44] reported that Tre application significantly improved sunflower growth by increasing antioxidant activities and nutrient uptake.

The application of BC increased the soil pH, creating favorable conditions for microbial activity and nutrient availability, resulting in increased growth and yield [45]. BC and Tre mediated an increase in pants’ ability to absorb nutrients like N, P, and K, which helped to mitigate adverse impacts of Pb. BC increased SOC, which improved nutrient-holding capacity and helped to mitigate the toxic effects of Pb stress on plants [32]. Co-application of BC and Tre reduced MDA and H_2_O_2_ concentrations by increasing osmolyte synthesis and antioxidant activity [33]. Our results indicate that BC enhances antioxidant activities. The uptake and concentration of Pb in plant tissues significantly increased; therefore, plants self-regulated antioxidants to mitigate the toxicity of Pb stress. The accumulation of HMs in plant tissues is a major route of their entry into the food chain [26]. BC and Tre decreased Pb accumulation in rice plants. The increased accumulation of Pb in plant tissues reduces nutrient uptake, leading to poor plant growth [34]. BC and Tre reduced Pb accumulation in plant parts by immobilizing Pb and decreasing the solubility of Pb in soil [46]. Trehalose also reduced Pb accumulation by forming Tre–Pb complexes, which reduced Pb accumulation in plant tissues. The exponential increase in plant growth caused by BC and Tre application was also linked with improved nutrient uptake and reduced Pb accumulation. These findings are consistent with the results of Zhang et al. [47], who reported that coconut shell BC decreased Pb uptake and accumulation in rice. Similarly, Wang et al. reported that Tre decreased Cd uptake and accumulation in rice by forming Tre and Cd complexes, leading to a reduction in Cd uptake and its accumulation [48].

The bio-concentration factor (BCF) is an important indicator to assess the accumulation of HMs in plant roots and shoots [49]. The results demonstrated that BC and Tre decreased both BCF and TF of Pb. Biochar and Tre reduced Pb availability and accumulation in plants, resulting in lower BCF and TF values for Pb. The application of BC increased soil pH because BC contains alkaline ions that increase soil pH [50]. BC increases soil pH, which facilitates the fixation and precipitation of HMs and reduces their availability [35]. BC application also increases SOC, which indirectly immobilizes Pb and decreases its availability and solubility [36]. The biochar-mediated increase in SOC also increased NPK availability, which reduced the toxicity of Pb via different mechanisms. For example, efficient uptake of N, P, and K by roots decreases Pb uptake, owing to the fact that the aforementioned nutrients are surrounded by Pb at uptake sites in roots. These nutrients also increase plant growth, which increases the plant’s ability to tolerate Pb stress [37]. In addition, these nutrients play essential roles in metabolism, including antioxidant production, which protects plants from Pb-induced toxicity [37].

## 4. Materials and Methods

### 4.1. Experimental Details

The study was conducted in the greenhouse at Chuxiong Normal University, Yunnan, China. The experimental site has a subtropical monsoon climate with an average annual temperature of 15.8 °C, annual rainfall of 873 mm, and annual shine of 2359 h. The soil was taken from a 0–20 cm layer of the experimental field and sieved to remove debris to fill the pots. A subsample of the soil was taken to determine diverse soil physicochemical properties [51]. The studied soil was silt loam with acidic pH (5.38), organic carbon content of 11.72 mg kg^−1^, total nitrogen (TN) content of 1.65 mg kg^−1^, and available phosphorus (AP) and available potassium (AK) contents of 27.12 and 109.11 mg kg^−1^, respectively. We analyzed the Pb concentration in the collected soil, using the digestion by solution (HNO_3_ and HClO_4_) and determined with atomic adsorption spectroscopy. The soil content of Pb was detected below the limit of 0.001 mg kg^−1^. Then, we applied 250 mg kg^−1^ exogenous Pb to the soil to achieve Pb stress. The following concentration of Pb aligns with the Risk Intervention Values for Soil Contamination of Agricultural Land in China (RIVSA, Pb ≤ 500 mg kg^−1^) specified for soil pH of 5.5–6.5 as outlined in GB 15618–2018. To prepare BC, maize straws were collected and pyrolyzed for 8 h at 600 °C [52]. Thereafter, BC was sieved (2 mm) and used to determine different characteristics. The BC had a significant amount of carbon (640 g kg^−1^), nitrogen (4.52 g), and alkaline pH (9.9).

### 4.2. Experiment Setup

**Figure 7:**
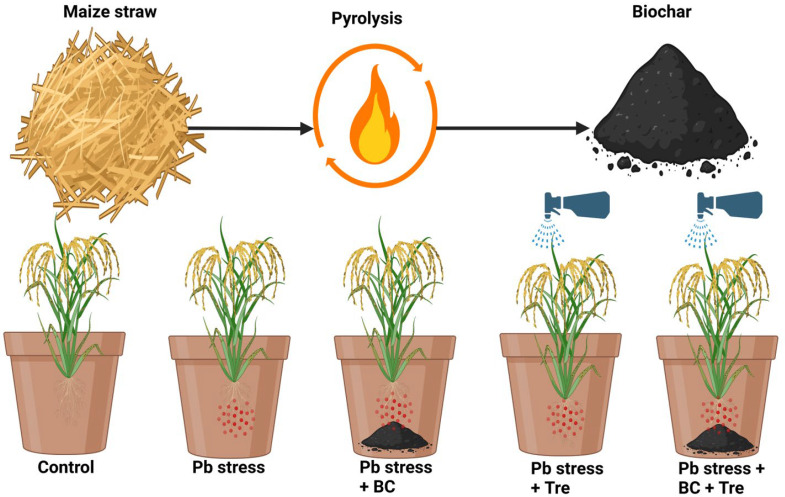
Flow chart of the experiment. The experiment was performed under five conditions as follows: control (without Pb stress and BC), Pb stress (250 mg kg^−1^), Pb stress (250 mg kg^−1^) + BC (2.5%), Pb stress (250 mg kg^−1^) + Tre (30 mM), and Pb stress (250 mg kg^−1^) + BC (2.5%) + Tre (30 mM).

The following five treatments were set up: control, Pb stress (250 mg kg^−1^), Pb stress (250 mg kg^−1^) + BC (2.5%), Pb stress (250 mg kg^−1^) + Tre (30 mM), and Pb stress (250 mg kg^−1^) + BC (2.5%) + Tre (30 mM) (Figure 7). The desired concentration of Pb was achieved by using Pb nitrate [(Pb (NO_3_)_2_)] [38]. Pb was added to the soil, which was processed for two months to stabilize the metals; thereafter, pots were filled with 8 kg of soil. For the BC treatments, the soil from each pot was removed, the BC was thoroughly mixed, and the pots were filled again. The rice cultivar “Nipponbare” used in this study was provided by Prof. Liu Li from Yunnan Agricultural University. Five seedlings (25 days old) were planted in each pot, and a 2–3 cm water level was maintained during the growing period. Foliar spraying of Tre was performed at the flag leaf stage via a hand sprayer, plant samples were taken after 25 days for further analysis, and the grain yield was recorded 95 days after transplanting. The samples collected for biochemical analyses were wrapped in aluminum foil and placed in liquid nitrogen until delivery to the laboratory for subsequent analyses. The experiment was repeated three times with 5 pots per treatment serving as replicates, and the experiment was performed under a completely randomized design.

### 4.3. Measurement of Plant Growth and Yield Traits

Rice plants were separated into roots and shoots to determine their lengths, as well as fresh and dry weights. The lengths of both plant parts were measured with a scale and weighed to determine the fresh weight. Later, samples were oven-dried (65 °C) to estimate dry weights. The plants were harvested to determine plant height (PH), tillers/pot (TPP), panicle length (PL), hundred-grain weight (HGW), grain and biomass yields, and harvest index (HI) with standard protocols.

### 4.4. Measurement of Chlorophyll Contents and Physiological Attributes

Leaf samples (0.5 g) were collected and extracted with acetone and concentrations of chlorophyll a, b, and carotenoids were estimated by methods of Lichtenthaler [53]. Fresh rice leaves were carefully collected, sealed in plastic, and weighed to determine fresh weight (FW) [54]. These samples were immersed in water for 24 h and weighed again (TW). Thereafter, leaves were dried, and RWC was estimated by the following equation: (FW − DW)/(TW − DW) × 100 [55]. For electrolyte leakage (EL), briefly, 0.5 g fresh leaves were submerged in water and placed in a water bath (40 °C) for 30 min, after which the first electrical conductivity (EC: EC_1_) was measured. The second EC (EC_2_) was taken by immersion of leaf samples in the water bath (40 °C) for 10 min, and the EL was estimated with the following protocol: EL% = (EC_1_/EC_2_) × 100 [54].

### 4.5. Estimation of Oxidative Stress Indicators and Osmolytes

The concentration of malondialdehyde (MDA) was estimated by extracting fresh samples (0.5 g) with 0.1% trichloroacetic acid (TCA) by adopting the procedures of Song, et al. [56]. The supernatant was collected after centrifugation (10,000 rpm; Eppendorf Centrifuge Model 5418) for 15 min, and the absorbance was read at 532 nm. To determine the H_2_O_2_ concentration, 0.5 g fresh samples were mixed with 1 mL of TCA (0.1%) solution and centrifuged (8000 rpm; Eppendorf Centrifuge Model 5418) for 15 min. Thereafter, 0.5 mL of supernatant was mixed with a reaction mixture containing 1 mL of potassium iodide (1 M) and 0.5 mL of potassium phosphate buffer (PPB: 10 mL), and the absorbance was read at 600 nm [57]. To estimate proline contents, briefly, fresh leaf samples (0.5 g) were homogenized with sulfosalicylic acid (3%) and centrifuged (10,000 rpm; Eppendorf Centrifuge Model 5418) for 10 min. The supernatant was incubated (100 °C) for 1 h after adding 2 mL of acid ninhydrin and glacial acetic acid. Then, the mixture was allowed to cool, toluene was added, and the absorbance was measured at 520 nm [58]. In the case of total soluble protein (TSP), fresh samples were collected and homogenized in PPB (5 mL). Thereafter, we centrifuged (14,000 rpm; Eppendorf Centrifuge Model 5418) the obtained extract for 15 min. Two milliliters of Bradford mixture was added to extract the absorbance, which was read at 595 nm [59].

### 4.6. Measurement of Antioxidant Activities

The antioxidant activities (APX, CAT, POD, and SOD) were estimated after plant samples were extracted with phosphate buffer [60]. Briefly, we extracted 0.5 g fresh leaf samples by using 6 mL of 50 mM cooled phosphate buffer solution (pH: 7.8). The extraction was performed in a precooled mortar and pestle following centrifugation (8000 rpm; Eppendorf Centrifuge Model 5418) for 20 min. The activity of APX was estimated at 290 nm with the methodology of Nakano and Asada [61], and CAT activity was assessed by the methods of Farman and Hadwan [62]. The POD activity was determined by measuring the rate of guaicol oxidation in the presence of H_2_O_2_, which was estimated using the methods of Van Doorn and Ketsa [63]. Finally, SOD activity was assessed by measuring the inhibition of 50% photochemical reduction in nitro-blue tetrazolium (NBT) at 560 nm [64].

### 4.7. Measurement of Nutrient Concentrations

The plant samples were collected and ground to make powder and digested using a mixture of acids (HCl and HNO_3_, 1:2) to determine the nutrient concentration in the tissues. The concentrations of calcium (Ca) and magnesium were determined by an Atomic Absorption Spectrophotometer (AAS) (GBC Australian, model no 932-AA), while a Flame Photometer Instrument (model FP910, PG Instruments Limited, Lutterworth, UK) was used to estimate the potassium (K) concentration [65]. The concentrations of nitrogen (N) and phosphorus (P) were determined using the Kjeldahl method and a spectrophotometer, respectively.

### 4.8. Assessment of the Pb Concentration in Soil and Plant and Analysis of Soil Physicochemical Properties

Rice plant samples were dried and digested with HCl and HNO_3_ (1:2), and Pb concentration was estimated using atomic absorption spectrophotometry. The soil pH was assessed with a pH meter, and soil N was estimated using the Kjeldahl method. Meanwhile, soil AP and AK concentrations were measured using sodium bicarbonate and ammonium acetate extraction methods, respectively [66]. The soil organic carbon content was estimated via the concentrated sulfuric acid external heating method. Moreover, the bio-concentration factor (BCF) and translocation factor (TF) were determined via the equations suggested by Malik, et al. [67].

(i) BCF = Pb concentration in roots/Pb concentration in soil
(ii) TF = Pb concentration in shoots/Pb concentration in roots

### 4.9. Statistical Analysis

The data for various traits were analyzed using one-way analysis of variance (ANOVA), and Tukey’s honestly significant difference (HSD) test was applied to determine significant differences between means at *p* < 0.05. The figures presented in the manuscript were generated with SigmaPlot 10. Principal component analysis (PCA) and correlation analyses were conducted using R Studio v.4.2.1.

## 5. Conclusions

Lead toxicity decreased rice growth and yield by impairing plant functioning and increasing Pb accumulation. Co-application of BC and Tre significantly increased photosynthetic pigments, antioxidant activities, osmolyte synthesis, and nutrient uptake, as well as decreased lead availability and oxidative damage, thereby leading to better growth and productivity. Biochar and Tre also decrease the translocation factor, Pb bio-concentration factor, and Pb bioavailability by increasing Pb immobilization and soil pH. These findings increase our understanding of using BC and Tre to mitigate Pb toxicity in rice and offer insights for safer rice production in Pb-polluted soils. This study was performed under controlled conditions; thus, long-term field studies need to be conducted in Pb-polluted soils to validate these findings. Moreover, in future studies, the effects of repeated BC and Tre applications over several growing seasons will be explored to determine the sustainability of this approach. In addition, we also aimed to perform spectroscopy studies to quantify Pb availability in plants and soil after applying BC and Tre. Moreover, in the future, molecular studies will be carried out to investigate the mechanisms by which BC and Tre induce Pb tolerance in rice plants.

## Figures and Tables

**Table 1:** Effect of biochar and trehalose on the oxidative makers, osmolytes, and antioxidant activities of rice under lead stress.

Treatments	EL (%)	MDA (mg/g FW)	H_2_O_2_ (mg/g FW)	Proline (mg/g FW)	TSP (mg/g FW)	APX (U/mg Protein)	CAT (U/mg Protein)	POD (U/mg Protein)	SOD (U/mg Protein)
Control	19.87 ± 1.32 d	3.44 ± 0.082 d	1.79 ± 0.10 e	0.623 ± 0.026 c	9.88 ± 0.78 a	5.30 ± 0.15 e	2.54 ± 0.070 e	0.180 ± 0.022 c	2.56 ± 0.09 e
Pb stress	49.33 ± 1.70 a	6.38 ± 0.17 a	4.69 ± 0.15 a	0.683 ± 0.20 bc	6.85 ± 0.56 c	5.94 ± 0.42 d	2.99 ± 0.086 d	0.237 ± 0.012 bc	3.10 ± 0.10 d
Pb + BC	23.63 ± 1.74 bc	4.04 ± 0.22 c	3.12 ± 0.82 c	0.850 ± 0.025 a	8.08 ± 0.12 b	6.86 ± 0.21 b	4.10 ± 0.084 b	0.377 ± 0.034 a	4.33 ± 0.08 b
Pb + Tre	25.30 ± 0.94 b	4.58 ± 0.22 b	3.45 ± 0.048 b	0.740 ± 0.029 b	7.34 ± 0.18 c	6.45 ± 0.70 c	3.65 ± 0.066 c	0.283 ± 0.025 b	3.93 ± 0.04 c
Pb + BC + Tre	22.20 ± 0.82 bc	3.64 ± 0.068 cd	2.75 ± 0.060 d	0.923 ± 0.020 a	8.56 ± 0.11 b	7.35 ± 0.42 a	4.49 ± 0.068 a	0.433 ± 0.017 a	4.78 ± 0.12 a

EL: electrolyte leakage, MDA: malondialdehyde, H_2_O_2_: hydrogen peroxide, TSP: total soluble protein, APX: ascorbate peroxidase, CAT: catalase, POD: peroxidase, SOD: superoxide dismutase. The data are presented as means ± SD (*n* = 3), and different letters indicate significance at *p* ≤ 0.05 level according to the Tukey HSD test. Here, control (without Pb stress and BC), Pb stress (250 mg kg^−1^), Pb stress (250 mg kg^−1^) + BC (2.5%), Pb stress (250 mg kg^−1^) + Tre (30 mM), and Pb stress (250 mg kg^−1^) + BC (2.5%) + Tre (30 mM).

**Table 2:** Effect of biochar and trehalose application on nutrient concentration of rice plant parts under lead stress.

Treatments	Root-N	Shoot-N	Root-P	Shoot-P	Root-K	Shoot-K	Root-Ca	Shoot-Ca	Root-Mg	Shoot-Mg
	mg·kg^−1^ DW									
Control	9.77 ± 0.32 c	12.61 ± 0.54 c	7.13 ± 0.7 cd	10.71 ± 0.37 c	16.66 ± 0.82 c	19.65 ± 1.23 d	46.63 ± 1.94 c	65.50 ± 0.94 c	35.40 ± 0.83 d	48.83 ± 0.95 d
Pb stress	8.70 ± 0.40 c	10.69 ± 0.38 d	6.14 ± 0.17 d	9.14 ± 0.90 c	14.23 ± 0.21 c	17.30 ± 0.43 d	42.90 ± 1.10 c	58.10 ± 1.90 d	31.37 ± 1.67 d	40.30 ± 0.78 e
Pb + BC	13.23 ± 0.33 b	18.14 ± 0.37 ab	9.87 ± 0.52 ab	13.97 ± 0.55 b	22.33 ± 0.97 b	30.37 ± 0.67 b	57.24 ± 1.68 b	77.47 ± 1.43 ab	51.47 ± 0.90 b	61.65 ± 0.63 b
Pb + Tre	11.95 ± 0.30 b	16.95 ± 0.28 c	8.59 ± 0.45 bc	12.40 ± 0.49 b	20.19 ± 0.82 b	27.03 ± 1.17 c	52.82 ± 1.25 b	72.70 ± 1.36 b	44.59 ± 2.47 c	56.40 ± 0.95 c
Pb + BC + Tre	15.17 ± 0.98 a	19.22 ± 0.45 a	11.37 ± 0.87 a	15.75 ± 0.73 a	26.84 ± 1.10 a	34.27 ± 0.86 a	64.67 ± 1.13 a	82.37 ± 1.68 a	57.19 ± 1.23 a	66.90 ± 1.69 a

N: nitrogen, P: phosphorus, K: potassium, Ca: calcium, and Mg: magnesium. The data are presented as means ± SD (*n* = 3), and different letters indicate significance at *p* ≤ 0.05 level according to the Tukey HSD test. Here, control (without Pb stress and BC), Pb stress (250 mg kg^−1^), Pb stress (250 mg kg^−1^) + BC (2.5%), Pb stress (250 mg kg^−1^) + Tre (30 mM), and Pb stress (250 mg kg^−1^) + BC (2.5%) + Tre (30 mM).

**Table 3:** Effect of biochar and trehalose on the soil properties after harvesting rice crop under lead stress.

Treatments	Soil Pb (mg kg^−1^)	Soil pH	AP (mg kg^−1^)	AK (mg kg^−1^)	TN (g kg^−1^)	SOC (mg kg^−1^)
Control	0.00 ± 0.00 e	5.39 ± 0.012 c	29.45 ± 0.73 a	109.67 ± 3.68 a	1.47 ± 0.034 a	23.73 ± 0.95 b
Pb stress	154 ± 3.86 a	5.40 ± 0.033 c	15.77 ± 0.54 e	63.94 ± 2.63 d	0.82 ± 0.024 d	23.03 ± 1.27 b
Pb + BC	109 ± 2.68 c	5.54 ± 0.021 b	20.20 ± 0.82 c	80.20 ± 1.63 bc	1.12 ± 0.021 bc	28.50 ± 0.70 a
Pb + Tre	125 ± 2.87 b	5.43 ± 0.017 c	18.07 ± 0.19 d	73.27 ± 0.98 c	1.03 ± 0.066 c	23.84 ± 0.49 b
Pb + BC + Tre	98 ± 2.37 d	5.62 ± 0.012 a	24.42 ± 0.68 b	84.93 ± 2.02 b	1.21 ± 0.025 b	31.10 ± 0.78 a

AP: available phosphorus, AK: available potassium, AN: available nitrogen, SOC: soil organic carbon. The data are presented as means ± SD (*n* = 3), and different letters indicate significance at *p* ≤ 0.05 level according to the Tukey HSD test. Here, control (without Pb stress and BC), Pb stress (250 mg kg^−1^), Pb stress (250 mg kg^−1^) + BC (2.5%), Pb stress (250 mg kg^−1^) + Tre (30 mM), and Pb stress (250 mg kg^−1^) + BC (2.5%) + Tre (30 mM).

## Data Availability

The raw data supporting the conclusions of this article will be made available by the authors, without undue reservation.
